# An automated method to establish patient specific asymmetric intrafraction motion monitoring tolerances for prostate radiotherapy

**DOI:** 10.1002/acm2.70507

**Published:** 2026-02-27

**Authors:** Bradley Beeksma, John Daniel, Erin Seymour, Andrew Dipuglia

**Affiliations:** ^1^ Department of Radiation Oncology Calvary Mater Newcastle Newcastle New South Wales Australia; ^2^ School of Information and Physical Sciences University of Newcastle Newcastle New South Wales Australia

**Keywords:** asymmetric motion management, dominant intraprostatic lesion, image‐guided radiotherap, intrafraction motion, patient specific tolerances, prostate radiotherapy, treatment planning automation

## Abstract

**Purpose:**

Intrafraction motion management is recognized as a critical component of radiotherapy. While clinical trials often recommend its use, they rarely define motion management tolerances. Consequently, institutions set tolerance levels independently, often without considering patient specific anatomy, dose distributions or direction of motion. This study introduces an automated method that can be used to derive patient specific, asymmetric intrafraction motion monitoring tolerances based on individualized treatment plans.

**Methods:**

Treatment plans for 20 prostate cancer patients receiving a simultaneous integrated boost to dominant intraprostatic lesions (DIL) were retrospectively analyzed. Referred to as the isocentre translation method, patient movement was simulated by recalculating plans with the isocentre shifted by 2, 4 and 5 mm in six different directions. The magnitude and direction that resulted in violations of dose constraints for organ at risk (OAR) rectum, bladder and urethra was determined assuming a systematic shift for all fractions. A second automated approach, the contour translation method, used Eclipse Scripting API to estimate directional tolerances via contour translation and Boolean operations, avoiding dose recalculation. Results for this method were validated against the isocentre translation method and compared for efficiency by assessing processing time.

**Results:**

The magnitude and direction of motion to cause OAR constraint violations were organ and patient specific. Urethral violations were most sensitive to shifts toward the DIL, whereas bladder and rectum constraints were primarily affected by anterior/superior and posterior shifts, respectively. The contour translation method was demonstrated to be equivalent to the isocentre translation method within an equivalence margin of 0.5 mm. The contour translation method significantly reduced processing time (4 min 40s vs. 47 min 52s per patient).

**Conclusion:**

The direction and extent of motion impacting OAR constraints vary by patient and organ, supporting the need for personalized intrafraction motion monitoring tolerances. The proposed contour translation method provides a practical, efficient process that is able to facilitate individualized motion management in clinical workflows.

## 1 INTRODUCTION

Dominant intraprostatic lesions (DIL) have been shown to be the main site of prostate cancer recurrence for patients who have relapsed locally following radiotherapy.^1^, ^2^ There are multiple clinical trials exploring the feasibility of dose escalation to the DIL with respect to clinical endpoints and patient toxicity, providing strong evidence for incorporation into standard practice for prostate radiotherapy.^1^, ^3^, ^4^, ^5^, ^6^, ^7^ Patients assigned to the trial arm of these studies receive a simultaneous integrated focal boost to the DIL, with the level of dose escalation limited by the constraints of nearby organs at risk (OAR), mainly the urethra, bladder or rectum. This dose escalation results in steep dose gradients within the plan, particular when an OAR is in close proximity to the DIL. However, since treatment plans are generated on a static 3D CT, they only represent a snapshot of the patient's anatomy in time. Consequently, potential intrafraction motion during treatment delivery and the potential impact on OAR toxicity is not always considered during the planning process.^8^, ^9^

There have been many studies that have assessed intrafraction prostate motion.^10^, ^11^, ^12^, ^13^, ^14^, ^15^, ^16^, ^17^ These studies show that the prostate exhibits positional variability, with greater displacement and magnitude over time. Movement occurs predominantly in the posterior and inferior directions, while lateral shifts are less pronounced. Anterior and superior shifts are typically brief and related to gas movement. Richter et al.^16^ also reported increased prostate displacement variance with time, noting that shorter treatment durations reduce the likelihood of intrafraction motion.

In the DIL dose escalated clinical trials reviewed by Poon et al.^8^ three of the 17 trials stipulated the use of intrafraction monitoring which has been deemed essential by Di Franco et al.^17^ when margins are less than 5 mm. In addition to this, TROG 18.01 phase III randomized clinical trial^18^ mandates a viable real time intrafraction motion management strategy to monitor for intrafraction prostate motion for the hypofractionated arms of the trial. Although the use of intrafraction monitoring is stipulated in these aforementioned studies, they typically do not specify what magnitude or direction of motion should prompt treatment interruptions, provide guidance as to how intrafraction motion management should be implemented, nor account for the impact of motion on OAR constraints. In the context of DIL boosts, patient motion during treatment in the direction towards the DIL boost, may have a significant impact on OAR toxicities due to the steep dose gradients surrounding DILs if tolerance settings are too large.^9^ Conversely, unnecessary treatment interruptions, as a result of tight tolerances, may result in an increase in treatment time if the motion occurred in a direction that would not impact the plan dosimetrically. Therefore, rather than using general, uniform based tolerances for intrafraction monitoring, a patient centered approach based on the individual's treatment plan, may be favorable. Patient specific tolerances that incorporate the directional dependence of dose gradients relative to OARs may be beneficial as individualized tolerances would eliminate ambiguity, ensure consistency across institutions, and provide clear, personalized monitoring tolerances for patients. This approach may also reduce unnecessary treatment interruptions by ensuring they occur only when motion negatively impacts plan dosimetry. Standardizing the approach of personalized intrafraction monitoring tolerances across institutions participating in clinical trials could subsequently improve the robustness of outcomes data by ensuring treatment delivery consistency between centers.

This study introduces an automated, efficient approach that can aid in the determination of patient specific, asymmetric intrafraction monitoring tolerances for prostate external beam radiotherapy. By quantifying the magnitude and direction of motion that would result in OAR dose constraint violations, this methodology provides a clinically meaningful framework for personalized intrafraction motion management. To the best of the authors’ understanding, this is the first study to propose such a methodology.

## 2 METHODS

Treatment planning data sets from 20 patients previously treated at Calvary Mater Newcastle for definitive prostate cancer were retrospectively identified. All patients had an identifiable DIL defined using prostate specific membrane antigen PET as well as T2 and Diffusion Weighted Imaging MRI sequences. Patients were prescribed a moderately hypofractionated dose regimen of 60 Gy in 20 fractions to the prostate and received a boost dose of up to 66 Gy to the DIL. A CTV to PTV margin of 5 mm was used for all patients. Focal boost dose to the DIL was maximized whilst still prioritizing departmental OAR constraints shown in Table 1. All patients were planned with a volumetric modulated arc therapy (VMAT) technique with two full arcs on a Varian Truebeam linac, utilizing either a 6 MV or 10 MV photon beam. Dose distributions were calculated with the AcurosXB v15.6.05 algorithm using the Eclipse (Varian Medical Systems, Palo Alto, USA) Treatment Planning System (TPS) with a 1.5 mm dose grid resolution. Due to dose escalation of the DIL, OARs were often planned to the limit of their dose constraint. To determine the magnitude and direction of patient movement that would lead to exceedance of the OAR dose constraints in Table 1, a planning study was performed.

**TABLE 1:** Departmental OAR constraints used for planning.

Structure	Constraint
Bladder Rectum	V60 Gy < 3%
	V57 Gy < 15%
	V54 Gy < 20%
	V46 Gy < 35%
	V38 Gy < 50%
Urethra	D0.3 cc < 61.8 Gy

**FIGURE 1:**
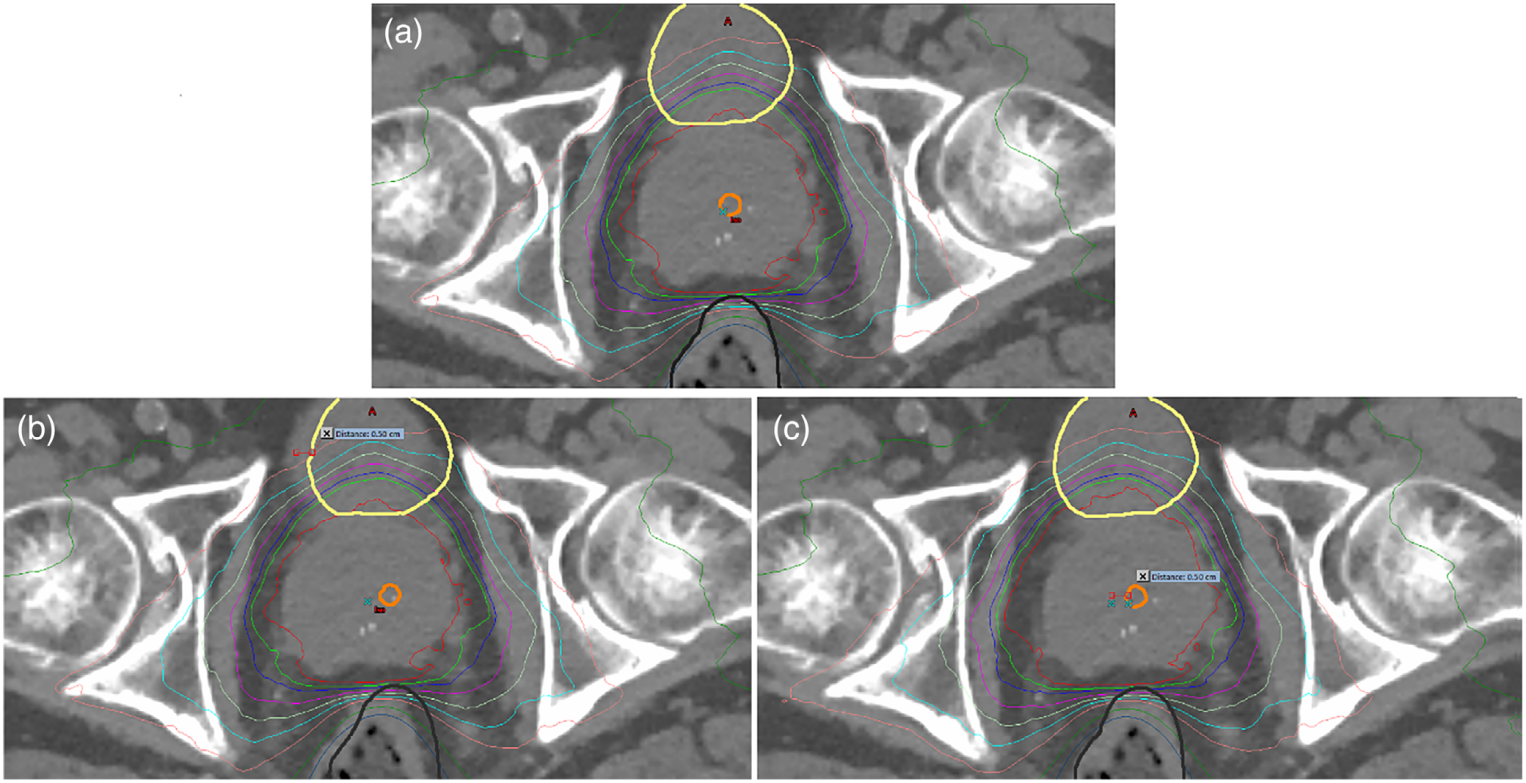
(a) Original plan dose distribution indicating bladder (yellow), rectum (black) and urethra (orange) contours. (b) Contour translation method, showing rectum, bladder and urethra OARs displaced 5 mm to patient left from their original position in A. (c) Isocentre translation method showing isocentre and dose displaced 5 mm to patient right from its original position in A.

Isocentre shift simulations are well established in the literature and have been adopted to study the dosimetric impact of positional errors on patient treatments.^19^, ^20^, ^21^ Since shifting the isocentre has the effect of displacing the dose distribution relative to the patient anatomy, this is a relative motion which can alternatively be thought of as moving the patient anatomy relative to the beam, thereby simulating patient motion. Repositioning and recalculating the beam at each discrete isocentre position is advantageous as this accurately reflects the dosimetry within the patients anatomy for any given isocentre position. However, this comes at a computational cost as dose must be recalculated per isocentre position. For each of the 20 patients, the original treatment plan (example in Figure 1a) was recalculated using the Eclipse batch plan uncertainty tool, with the isocentre shifted 2, 4, and 5 mm in each of the superior, inferior, left, right, anterior, and posterior directions. The magnitude of these shifts were chosen as a trade–off between calculation time and accuracy of data interpolation within the dose grid, with a maximum 5 mm translation chosen to represent the CTV to PTV margin. Including the original plan without shifts, this approach resulted in the generation of 19 distinct treatment plans for each of the 20 patients. For each of these plans, the dose volume histogram (DVH) was recalculated and exported. A custom Python script was developed to extract dose values for the urethra D0.3 cc, rectum/bladder V60 Gy, and V57 Gy from each treatment plan. The lower dose constraint values of 54, 46, and 38 Gy in Table 1 were chosen to be excluded from the analysis, as these constraints are infrequently exceeded in clinical plans. Linear interpolation was used to determine the submillimeter magnitude of isocentre shifts that would result in the violation of each organ at risk (OAR) constraint for all directions. A maximum shift of 5 mm was selected for investigation, as this corresponds to the largest tolerance applied clinically, as dictated by the 5 mm CTV to PTV margin. Consequently, if either method did not indicate an OAR constraint violation for shifts up to and including 5 mm, the shift reported for that method was assigned a value of 5 mm, thereby ensuring that CTV coverage would be maintained during treatment. The rationale of this threshold is consistent with the approach given by Wilkinson et al.^22^ The method of shifting the isocentre, and recalculating the dose distribution to determine the magnitude and direction to violate each OAR constraint will henceforth be referred to as the “isocentre translation method” and is illustrated in Figure 1c.

While the isocentre translation method offers an accurate and robust means of assessing dose distributions as a function of simulated patient motion, there are notable limitations to its use. Manually recalculating treatment plans is labor intensive and results in the generation of substantial amounts of data, necessitating the export of dosimetric parameters to external custom software for analysis. Incorporating these extra steps into the local clinical work flow is unfeasible due to the increase in time required per patient compared to the standard workflow using the features available in the Eclipse TPS. To address these limitations, a custom Microsoft .NET application script was written in C# leveraging the Eclipse Scripting Application Programming Interface (ESAPI). The script determines the magnitude and direction of an OAR's movement, which would result in a violation of the DVH constraints without requiring dose recalculation, allowing individualized tolerance determination to be performed efficiently within the TPS. For the urethra, the script begins by converting the 61.8 Gy isodose line into a structure. The urethra contour is then copied into a temporary structure and translated by 1 mm in one direction. The overlapping volume between this temporary translated structure and the 61.8 Gy isodose structure is calculated using the Boolean ‘And’ operator. If the overlapping volume exceeds the OAR constraint for the urethra as specified in Table 1 (0.3 cc), the script records the direction and magnitude of the translation. If the constraint is not exceeded, the script increases the translation of the temporary structure in 1 mm increments until either the constraint is exceeded or an expansion of 5 mm is reached. This process then repeats, performing the translation in each of the superior, inferior, left, right, anterior, and posterior directions. Subsequently, the same process is applied to both the rectum and bladder, calculating the percentage overlap of the OAR in 1 mm translation increments for both the 60 and 57 Gy isodoses, with the thresholds based on the constraints specified in Table 1. At the end of this process, the script outputs the magnitude and direction at which each OAR constraint was exceeded. Linear interpolation between adjacent DVH points is employed to calculate the submillimeter shift at which the specified constraint was exceeded. Using the same rationale as the isocentre translation method, if the constraint is not exceeded, the magnitude for that direction is set to 5 mm. Using Boolean operations on contours is advantageous in that it is significantly faster and produces substantially less temporary data to manage compared to shifting and re‐calculating treatment plans. The method of assessing OAR doses with contour translation is illustrated in Figure 1b and will henceforth be referred to as the “contour translation method.” In essence, the isocentre translation method shifts the isodose relative to the patient, whereas the contour translation method shifts the contour relative to the isodose distribution. Since the contour translation approach does not involve dose recalculation, it does not capture changes in beam geometry, attenuation, or scatter associated with isocentre displacement.

The required translational magnitudes to exceed each OAR constraint with the contour translation method were compared with those values obtained using the isocentre translation method which was considered the ground truth. The difference between the two methods (contour translation—isocentre translation) was then calculated per patient per direction to validate the contour translation method for accuracy.

Agreement between the contour translation and isocentre translation methods was evaluated using a paired *t*‐test and the Two One‐Sided Tests (TOST) procedure. The paired *t*‐test was employed to assess the presence of systematic bias between the two methods at a significance level of *α* = 0.05. The null hypothesis stated that the mean difference between the distances reported by the contour translation and isocentre translation methods was equal to zero, while the alternative hypothesis stated that the mean difference was significantly different from zero.

The TOST procedure was used to evaluate whether the distances reported by the contour translation and isocentre translation methods were statistically equivalent within a predefined margin. The TOST method involves performing two one‐sided *t*‐tests: one testing whether the mean difference exceeds the upper equivalence bound, and the other testing whether it falls below the lower bound. The null hypothesis stated that the true mean difference lay outside the equivalence bounds, whereas the alternative hypothesis stated that it lay entirely within these bounds. Equivalence was concluded only if both one‐sided tests were significant at *α* = 0.05, thereby rejecting the null hypothesis of non‐equivalence and indicating that the two methods were statistically equivalent within the specified margin. An equivalence margin of ± 0.5 mm was selected, corresponding to approximately half the in‐plane pixel dimensions and one‐quarter of the slice thickness of the CT datasets used in this study. A narrower margin would fall well below the spatial resolution limits of the imaging utilized for these patients and would therefore be considered clinically insignificant.

To assess the efficiency of each methodology, a timing analysis was performed on a representative subset of three patients. The recorded time corresponded to the duration required to complete the full process for each method in determining the shift at which each OAR constraint was exceeded.

## 3 RESULTS

**FIGURE 2:**
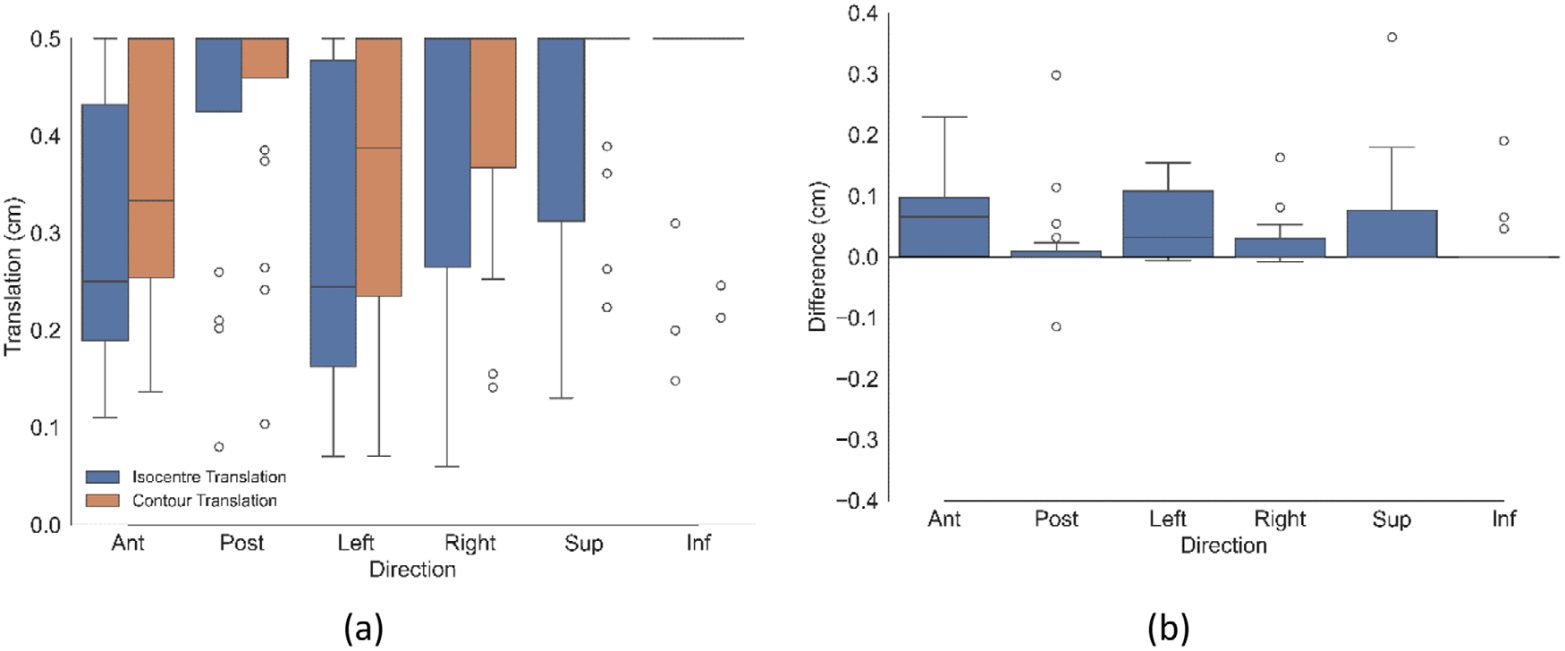
(a) Magnitude of shifts resulting in violations of urethra dose constraint for both contour and isocentre translation methods for all 20 cases. (b) Difference between the two methods (contour translation—isocentre translation).

**FIGURE 3:**
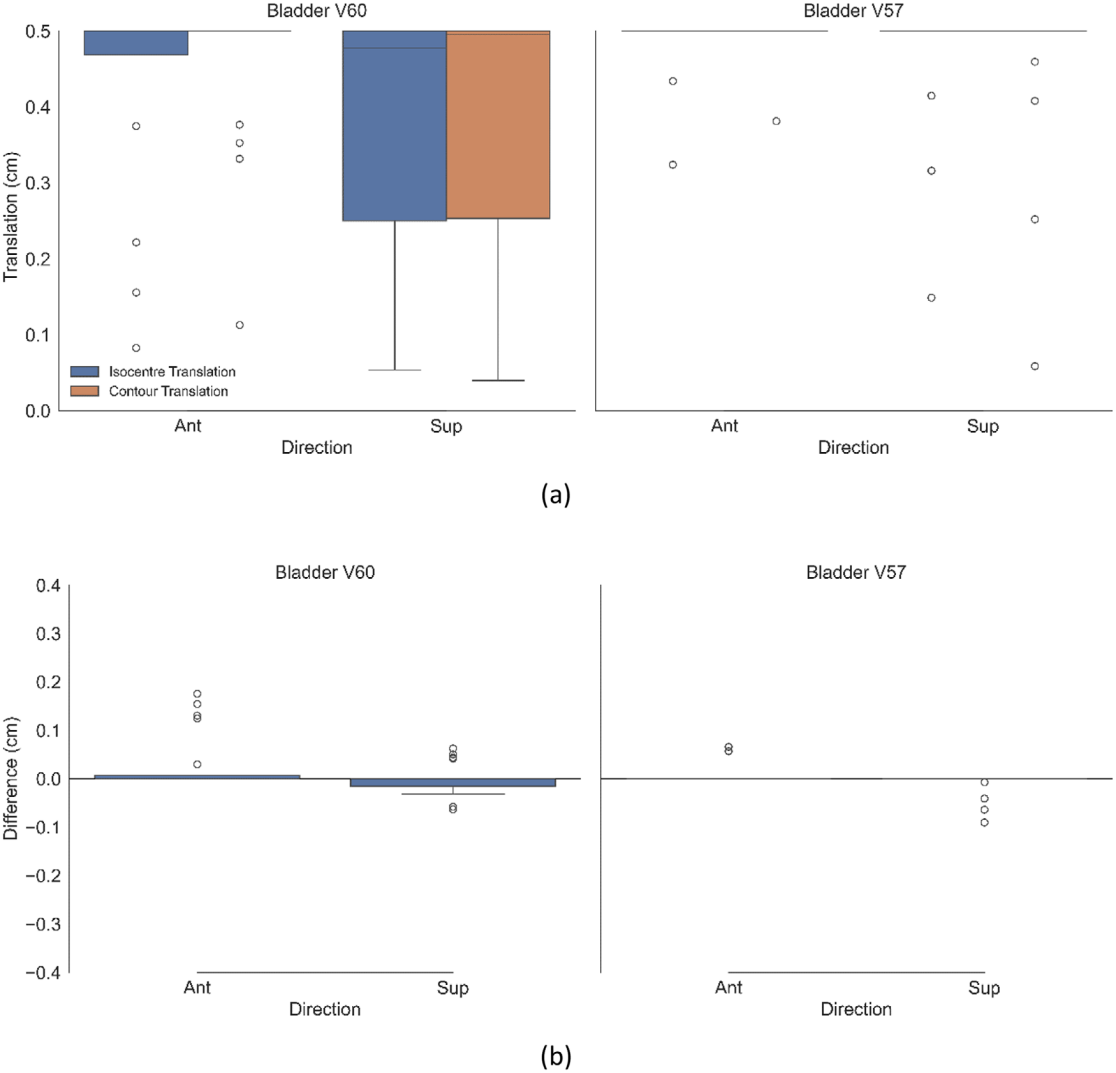
(a) Magnitude of shifts resulting in violations of bladder dose constraint for both contour and isocentre translation methods for all 20 cases. (b) Difference between the two methods (contour translation—isocentre translation).

**FIGURE 4:**
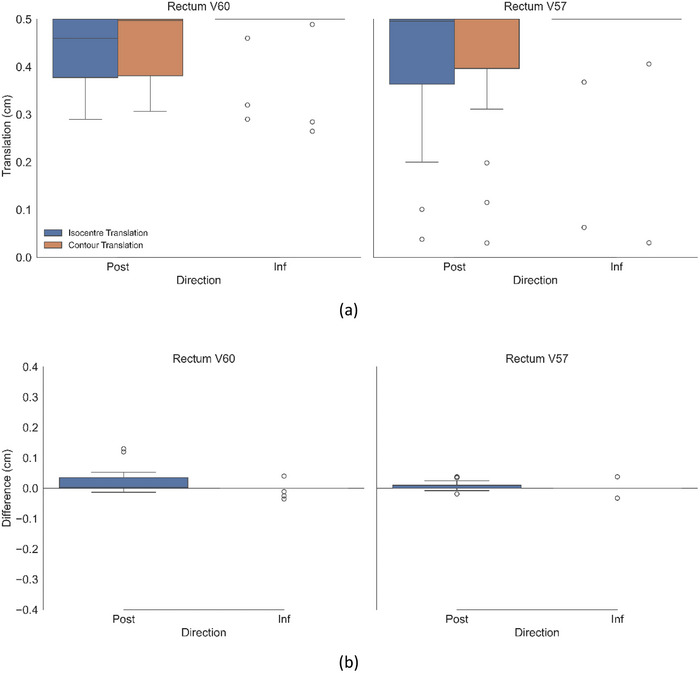
(a) Magnitude of shifts resulting in violations of rectum dose constraint for both contour and isocentre translation methods for all 20 cases. (b) Difference between the two methods (contour translation—isocentre translation).

Panel a of Figures 2, 3, 4 display box plot distributions of translations required to exceed OAR constraints in each direction for each patient for both the contour and isocentre translation methods. Urethral DVH violations were noted for all directions. Bladder DVH violations were only exceeded with shifts in the anterior and superior directions, while rectal DVH constraints only exceeded with shifts in the posterior and inferior directions. The directions where no constraints were exceeded have been omitted from their respective figures for simplification.

**FIGURE 5:**
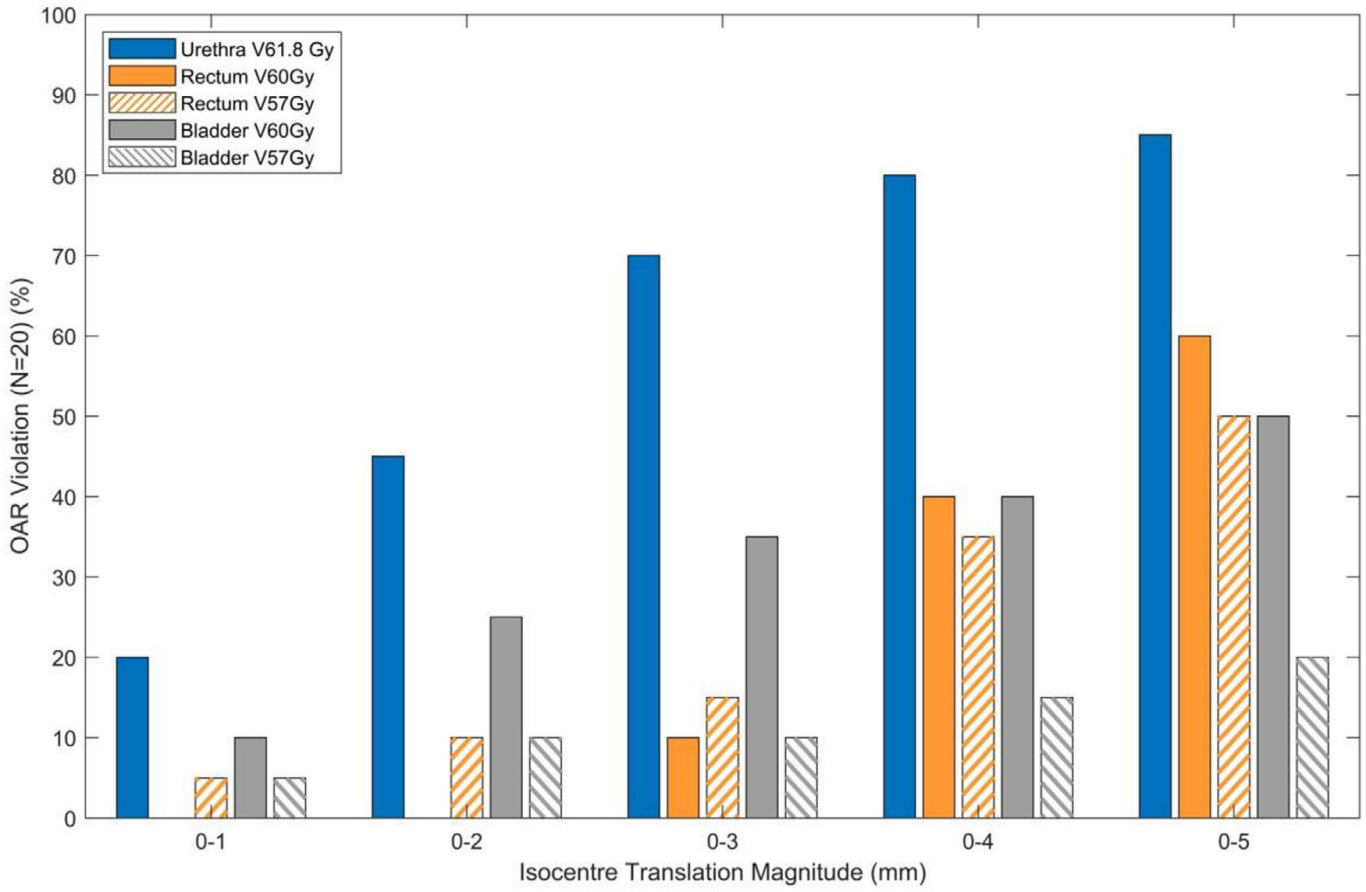
Percentage of patients that exceeded OAR DVH constraints for a given magnitude of simulated patient movement as determined by the isocentre translation method.

Panel b of Figures 2, 3, 4 shows the difference between the box plots shown in panel a. Per patient, panel b therefore specifies the difference in magnitude between the two methods (result of the contour translation method minus the isocentre translation method). Figure 5 shows the proportion of patients exceeding an OAR DVH constraint as a function of discrete isocentre translational shifts without segregation by the specific anatomical direction.

Paired *t*‐tests and equivalence testing results are summarized in Table 2. Presented are the results of the paired *t*‐test, including the mean difference ± standard deviation (SD), sample size (*n*), degrees of freedom (df), *t*‐statistic (*t*), *p*‐value, 95% confidence interval (CI) for the mean difference, as well as the TOST *p*‐values and equivalence conclusion for each OAR. Although the urethra and rectum showed statistically significant mean differences with *p* < 0.05, all OAR results met the predefined equivalence criteria of 0.5 mm.

**TABLE 2:** Paired *t*‐test and TOST equivalence results of comparing isocentre and contour translation methods using an equivalence margin of 0.5 mm. Standard deviation (SD), sample size (*n*), degrees of freedom (df), *t*‐statistic (*t*), 95% confidence interval (CI).

OAR	Mean diff (mm) ± SD	*n*	*t*(df)	*t*‐test *p*‐value	95% CI (mm) Lower / Upper	TOST *p*‐value	Equivalence conclusion
Urethra	0.4 ± 0.7	120	−6.15 (119)	<0.001	0.25 / 0.49	both < 0.001	Equivalent
Rectum	0.0 ± 0.1	240	−2.51 (239)	0.012	0.01 / 0.04	both < 0.001	Equivalent
Bladder	0.0 ± 0.2	234	−1.45 (233)	0.150	−0.01 / 0.05	both < 0.001	Equivalent

The timing performance exhibited minimal variability across different patients. Presented in Table 3, the contour translation method demonstrated an almost tenfold improvement in speed compared with the isocentre translation method, corresponding to a 90% reduction in the time required to determine the shift magnitude needed to exceed the DVH constraint. This can be attributed to the automated nature of the script which was embedded as an executable within Eclipse compared to the dose calculation time requirement for 18 plans and extraction of DVH metrics from these plans for comparison.

**TABLE 3:** Total time taken to determine patient specific asymmetric intrafraction motion tolerance using each methodology averaged over three patients.

Contour translation method	Isocentre translation method
4 min 40s	47 min 52s

## 4 DISCUSSION

By recalculating and analyzing treatment plans under simulated patient positional shifts, this study provides insight into the dosimetric impact of intra fractional prostate motion on the urethra, bladder, and rectum for patients with a DIL. The urethral constraint was demonstrated to be the most sensitive to positional shifts. As shown in Figure 2, no consistent directional dependence was observed for the urethra across the 20 patient plans. These findings were attributed to the variable spatial location of the DIL within the prostate capsule across the patient cohort. Since the urethra is positioned centrally within the prostate, the dose it receives is strongly influenced by its proximity to the DIL. Analysis of individual patient data revealed that directional shifts toward the DIL boost region would result in urethral constraint violations, whereas shifts away from the DIL boost did not. While there was no general directional dependence shown across the patient cohort, a patient specific directional dependence was evident, correlated by the spatial relationship between the DIL and the urethra. Unlike the urethra, both rectal and bladder constraints demonstrated a marked directional dependence. As shown in Figure 3, rectal constraints were primarily violated with posterior shifts, with only a marginal number of violations occurring with an inferior shift and none in other directions. This pattern was consistent for both the V60 Gy and V57 Gy constraints. Similarly, Figure 4 shows the bladder being violated predominantly with anterior shifts, with a minimal number of violations recorded for the superior direction and none for the other directions. These results can be attributed to the anatomical positions of the OARs relative to the target, with the rectum located posterior to the prostate and the bladder situated anatomically anteriorly and superior. Consequently, patient movement in these directions moves the OARs towards the higher dose region of the prostate, resulting in constraint violations. These findings highlight the importance of not only the magnitude but also the direction of intrafraction patient motion.

As summarized in Table 2, statistically significant differences between the two methods was demonstrated for the urethra and rectum with *p*‐values of < 0.001 and 0.012 respectively. These differences however are small in magnitude and were shown to be statistically equivalent within the equivalence bound of ± 0.5 mm when evaluated using the TOST. Across all OARs, the contour translation method systematically reported that larger positional shifts were required to induce an OAR constraint violation compared to the isocentre translation method. This is indicated by the positive values of the mean difference and negative *t*‐statistic indicated in Table 2, as well as being visually evident in panel b of Figures 2, 3, 4 with the difference between the two methodologies being systematically positive. This may be attributed to the fundamental differences between the two methodologies. With the isocentre translation method, the entire dose matrix is recalculated with each iteration. Consequently, at each isocentre position, the source to surface distance changes, and the radiation beam interacts with a shifted region of the patient's anatomy, thereby modifying the path length, attenuation, and scatter characteristics that influence the calculated dose distribution. Conversely, the contour translation method translates the contour through the original static dose matrix and dose is not recalculated. Although the contour translation method overestimates the shifts which would result in OAR dose constraint violations relative to those reported by the isocentre translation method, equivalence testing reinforces that these differences are unlikely to be clinically significant, as the reported shifts between the two methods were determined to be equivalent within 0.5 mm, which is substantially smaller than the spatial resolution of the imaging used for these patients (∼1 mm in‐plane pixel dimensions and 2 mm slice thickness). These results support the practical interchangeability of the two approaches.

Wilkinson et al.^22^ reported treatment times for a similar patient cohort being 05:06 ± 1:19 minutes for patients treated with no intrafraction interventions. Curtis et al.^23^ demonstrated that over a four minute time period, 5% of patients exhibit intrafraction motion ≥3 mm where Di Franco et al ^17^ reported that 16% of patients exhibit a similar magnitude of intrafraction motion over a comparable timeframe. Figure 5 illustrates the percentage of plans in this study that would have exceeded the DVH constraint for a given magnitude of movement. For a shift of ≤3 mm, constraint violations occurred for the urethra (V61.8 Gy), rectum (V60 Gy, V57 Gy), and bladder (V60 Gy, V57 Gy) in 70%, 10%, 15%, 35%, and 10% of cases respectively. These results highlight that some patients may still experience OAR DVH constraint violations for relatively small shifts over a treatment course and strengthens the argument for the necessity of personalized directional dependent intrafraction motion monitoring tolerances as has been discussed in the literature.^17^ The contour translation method was thus developed to create a feasible, reliable way to generate patient specific tolerances and address the labor intensive nature of the gold standard isocentre shift method. By leveraging ESAPI, the script operates directly within the TPS, eliminating the need for external data export. Ensuring the process is executed from within a single treatment plan removes the most significant time investment associated with the isocentre translation method which is needing to generate and recalculate additional plans for each shift required. This therefore also eliminates the generation of excessive data and streamlines the evaluation process. The efficiency gain was quantified by the timing study which showed a significant speed improvement using the contour translation method with almost a ten‐fold decrease in time compared to the isocentre translation method. Although calculation times for the isocentre translation method will differ across institutions due to variations in hardware performance and computational infrastructure, with a total execution time of less than 5 minutes, the contour translation method timing is feasible for use in a busy clinic. Although this study used the Varian Eclipse TPS, the methodology described is vendor agnostic and applicable to any other TPS capable of script based contour manipulation.

As recommended by Tudor et al ^24^ pre‐determined tolerances for systems that track motion during treatment to account for non‐periodic motion can be used in CTV to PTV margin calculations. They however do not provide guidance how to determine these tolerances. The contour translation method provides a solution that can be used to aid in the determination of intra fraction motion management tolerances for systems designed to track implanted gold fiducials for prostate radiotherapy as it ensures OAR DVH constraints are respected by considering individual patient planned dose distributions. It could also be utilized by institutions that do not employ planning organ at risk volumes (PRVs) within their planning process to assess the robustness of treatment plans. It does not replace, nor does it account for, other uncertainties associated with radiotherapy, such as contouring or image fusion when defining CTV to PTV margins. While validated for moderately hypofractionated prostate cancer regimens, this approach is likely also relevant to accelerated dose regimens in the prostate and other treatment sites, such as the liver. In these settings, intrafraction motion management using fiducials is common, and anatomical shifts during treatment may have greater clinical impact. Although fiducial based motion management was the focus of this study, the methodology could be applied to any intrafraction motion management system that allows asymmetric tolerances. Although the contour translation method has been demonstrated to be useful in an anatomical region where OARs are likely to shift relative to targets, it may also be feasible for more rigid anatomical locations, such as the brain or spine. In these sites with serial OAR, motion exhibits significant asymmetric dosimetric impact, with movement being far more critical in certain directions than others. Although the applicability and accuracy of this method would need to be evaluated for their specific use case. The methodology would not be applicable to systems such as surface guided motion monitoring, which cannot track internal organ motion.

The contour translation method offers the potential to standardize intrafraction motion management protocols across clinical trial centers, facilitating patient specific monitoring tolerances. This approach could help minimize unnecessary treatment interruptions while optimizing OAR protection, particularly for prostate treatments, where posterior margin reduction is commonly included despite evidence that this direction exhibits the greatest magnitude and likelihood of motion^10^, ^11^, ^12^, ^13^, ^14^, ^15^, ^16^, ^17^ Future work could be extended to include rotational shifts, which may better reflect typical patient motion patterns.

## 5 Conclusion

This study quantified the magnitude and direction of patient specific positional shifts that would result in urethral, bladder, and rectal dose constraints violations in prostate radiotherapy with a DIL boost. An automated method to determine these positional shifts utilizing contour translation was developed and validated against the conventional isocentre translation and dose calculation method. The automated method demonstrated dosimetric equivalence within a 0.5 mm margin, while achieving a tenfold reduction in processing time. This efficiency supports the method's suitability to provide a practical and efficient means of generating patient specific tolerances suitable for clinical integration.

The urethra was the most motion sensitive OAR, with directional dependence varying between patients according to the spatial relationship between the urethra and the DIL. In contrast, bladder and rectal violations exhibited consistent anatomical directional dependence, predominantly anterior/superior and posterior shifts, respectively. These results highlight that both the magnitude and direction of motion are critical when determining intrafraction motion management tolerances. The contour translation method provides a practical solution for generating patient specific, asymmetric motion monitoring tolerances tailored to individual anatomy and planned dose distributions. This approach offers the potential to optimize OAR protection and standardize the use of individualized intrafraction motion management tolerances in prostate radiotherapy.

## AUTHOR CONTRIBUTIONS

**Bradley Beeksma (Primary Author**): Had the original idea for the works, designed software infrastructure and code development. Performed some data interpretation and analysis of results. Primarily wrote the manuscript. **John Daniel**: Performed data collection, analysis and interpretation. Designed analysis software. **Erin Seymour**: Contributed to project planning and interpretation of results as well as writing of the manuscript. **Andrew Dipuglia**: Assisted methodology design, conducted data analysis and interpretation, and contributed to the writing of the manuscript. All authors reviewed and approved the final manuscript, agreeing to be accountable for the work's accuracy and integrity.

## CONFLICT OF INTEREST STATEMENT

The authors declare no conflicts of interest.
